# Hypofractionated Radiotherapy in Combination With Chemotherapy Improves Outcome of Patients With Esophageal Carcinoma Tracheoesophageal Groove Lymph Node Metastasis

**DOI:** 10.3389/fonc.2020.01540

**Published:** 2020-08-28

**Authors:** Jian Wang, Jingping Yu, Youqin Jiang, Dong Pei, Haiwen Zhu, Jianlin Wang

**Affiliations:** ^1^Department of Radiotherapy, Jiangyin People’s Hospital, Jiangyin, China; ^2^Department of Radiotherapy, The Affiliated Changzhou No. 2 People’s Hospital, Nanjing Medical University, Changzhou, China; ^3^Department of Radiotherapy, Yancheng No. 3 People’s Hospital, Yancheng, China

**Keywords:** esophageal cancer, tracheoesophageal groove lymph node metastasis, hypofractionated radiotherapy, conventional fractioned radiotherapy, chemotherapy, prognosis

## Abstract

This study investigated the efficiency and safety of hypofractionated radiotherapy (HFR) combined with paclitaxel chemotherapy for the treatment of postsurgery tracheoesophageal groove lymph node (TGLN) metastasis in patients with esophageal cancer (EC). Fifty-three EC patients with TGLN metastasis after surgery admitted to the Yancheng Third People’s Hospital from January 2013 to June 2015 were included in this study. They were randomly divided into the HFR group (*n* = 25) and conventional fractioned radiotherapy (CFR) group (*n* = 28) based on the random grouping method. Patients in the HFR group received treatment with radiation of 60 Gy (5 fractions per week, total 20 fractions) combined with paclitaxel chemotherapy at a dose of 50 mg once per week for 4 weeks. Patients in the CFR group received radiation of 60 Gy (5 fractions per week, total 30 fractions) combined with paclitaxel chemotherapy at a dose of 50 mg once per week for 6 weeks. The adverse events and treatment outcomes in these two groups were analyzed. It was found that there was no significant difference in the incidence of radiation esophagitis in the HFR group compared with that of the CFR group (grades 3–4, 44.0 vs. 25.0%; *P* = 0.149). There was no statistical difference in the incidence of radiation pneumonitis between these two groups (grades 3–4, 16.0 vs. 7.1%; *P* = 0.314). No statistical difference was noticed in complete response (CR), partial response (PR), and no response (NR) between these two groups. The median overall survival (OS) in the HRF group was significantly longer compared with that of the CRF group (24.2 months (95% CI, 16.2–32.1 months) vs. 11.8 months (95% CI, 9.2–14.4 months); *P* = 0.024). Our results indicated that the combination of HFR and chemotherapy improved the prognosis of EC patients with TGLN metastasis with no increased adverse events.

## Introduction

Esophageal cancer (EC), the common malignancy in China, is one of the leading causes of cancer-related death (1). Currently, surgery is preferred for the treatment of EC; however, a larger number of patients may present tracheoesophageal groove lymph node (TGLN) metastasis after surgery, with an incidence of 12–80% (2–5). Most of these patients are featured by hoarseness, bucking, and dyspnea, which hamper their quality of life. Furthermore, TGLN is also a negative prognostic factor for patients with EC. Radiotherapy is a major treatment option for TGLN metastasis in EC patients (6). Recently, the application of intensity-modulated radiation therapy (IMRT) contributes to target conformance and dose homogeneity, which reduces the dose of radiation to normal tissues and increases the local control and survival rate (7).

In a previous study, the efficiency and prognosis of EC patients who underwent chemoradiotherapy (CRT) were superior to that of the radiation monotherapy, and the related adverse events were tolerable (8). In clinical practice, conventional fractionated radiotherapy (CFR) has been commonly used for the treatment of EC. In recent years, it has been reported that hypofractionated radiotherapy (HFR) is feasible for treating moderate and advanced EC as it contributes to the overall survival (OS) (9, 10).

In this study, we investigate whether HFR could be used to improve the treatment efficacy in EC patients with TGLN metastasis after surgery. EC patients with TGLN metastasis were divided into two groups and treated with HFR or CFR, combined with paclitaxel chemotherapy. The treatment efficiency, toxicity, and prognosis of patients who underwent these two different treatment options were analyzed.

## Materials and Methods

### Patients

Postoperative EC patients with TGLN metastasis admitted to the Yancheng Third People’s Hospital from January 2013 to June 2015 were included in this study. The inclusion criteria were as follows: (1) patients aged less than 75 years, with clinical symptoms of hoarseness and bucking, after excluding vocal cord lesions; (2) patients who were diagnosed with TGLN metastasis using CT, MRI, and/or PET-CT; (3) patients with no severe cardiovascular diseases (CVDs), renal or splenic dysfunction; and (4) patients with an expected survival duration of at least 3 months. The exclusion criteria were as follows: (1) women with pregnancy or lactation; (2) patients complicated with concurrent malignancies; (3) patients with metastasis in the other organs and/or lymph nodes; (4) patients with CRT contraindications; and (5) patients with inadequate follow-up data. The following variables were gathered for analysis: age, gender, previous history, clinical symptoms, laboratory test results, and imaging findings. All participants provided informed consent. The study protocol was approved by the Ethical Committee of Yancheng Third People’s Hospital. This clinical trial was registered in the Chinese Trial Registry (ID: CTR1800016848).

### Grouping of Patients

As the radiation frequency in both groups was different in this study, we could not arrange the blinded study. Instead, an open-label study was conducted. The patients were randomly divided into two groups: the HFR group and the CFR group. Patients in the HFR group received radiation of 60 Gy/20 fractions (3 Gy/fraction, 5 fraction/week) combined with chemotherapy using paclitaxel with a dose of 50 mg once per week for 4 weeks. Patients in the CFR group received radiation of 60 Gy/30 fractions (2 Gy/fraction, 5 fraction/week) combined with chemotherapy using paclitaxel with a dose of 50 mg once per week for 6 weeks.

For the radiotherapy, all the patients were fixed with a thermoplastic head mask, followed by cervical and thoracic scanning with a slice thickness of 2.5 mm. The CT images were delivered to the Eclipse system. The gross tumor volume (GTV) was defined as TGLN shown on CT, MRI, and/or PET-CT scans. Planning target volume (PTV) was termed by adding a 1-cm margin around the GTV. The maximal doses for the spinal cord in the CRT and HRT groups were less than 45 and 40 Gy, respectively. The average dose for lung in the CRT and HRT groups were less than 13 and 10 Gy, respectively. The volume of the whole lung receiving ≥20 Gy (V_20_) in the CRT and HRT groups was less than 25 and 20%, respectively. The treatment was carried out in a linear accelerator (Varian Unigue) using a photon beam of 6 MV. For the chemotherapy, paclitaxel was given via intravenous drip (50 mg) before radiotherapy for 4 weeks in the HFR group and 6 weeks in the CFR group.

### Patients Follow-Up

The evaluation for the acute radiation-induced esophageal and/or pulmonary injury was based on the standards proposed by the Radiation Therapy Oncology Group (RTOG) in 1997 (11). The lymph node metastasis was evaluated using the Response Evaluation Criteria in Solid Tumors (RECIST, version 1.1). After treatment, the patients were followed up every 3 months within the first 2 years and 6 months once after 2 years. The follow-up data included case history, physical examination, laboratory test results, electrocardiogram, cervical CT, and thoracic CT. The primary endpoint was OS. All the patients were followed up until November 30, 2017.

### Statistical Analysis

The SPSS 19.0 software was used for the statistical analysis. Measurement data were presented as mean ± standard deviation and were compared using the Student’s *t* test. The numeration data were compared using the *Chi* square test. Non-parametric statistics was used for the analysis of ranked data. *Kaplan-Meier* method was used to calculate the OS and PFS. *Log rank* test was used for the analysis of prognosis, and *Cox* regression analysis was used for the multivariate analysis. *P* < 0.05 was considered to be statistically significant.

## Results

### Patients’ Characteristics and Adverse Events of Radiotherapy

Fifty-three patients were included in the study. Among them, 25 patients were in the HFR group and 28 in the CFR group. There was no statistical difference in the demographic characteristics of patients, such as sex, age, site of lymph nodes, diameter of lymph node, and clinical TNM stage between the two groups (*P* > 0.05; Table 1).

**TABLE 1:** Patients’ characteristics.

Variables	*N*	Test group (*n* = 25)	Control (*n* = 28)	χ^2^ value	*P* value
Sex				0.007	1.000
Male	40	19	21		
Female	13	6	7		
Age (years)				1.002	0.365
37–59	39	20	19		
60–75	14	5	9		
Site of lymph nodes				–	0.856
Left	28	12	16		
Right	19	10	9		
Both	6	3	3		
Diameter of lymph nodes (cm)				0.335	0.769
≤2	17	9	8		
>2	36	16	20		
T stage				–	0.784
T1–2	16	7	9		
T3	31	16	15		
T4	6	2	4		
N stage				00.061	1.000
N0	14	7	7		
N1	39	18	21		
TNM stage				–	0.452
I	3	2	1		
II	36	15	21		
III	14	8	6		

The adverse events of radiotherapy for patients with EC were mainly manifested by radiation esophagitis and pneumonitis. HFR caused no alternation to the incidence of radiation esophagitis and pneumonitis compared with CFR. The incidence of grades 1–2 or 3–4 radiation esophagitis in the HFR group showed no statistical difference compared with that of the CFR group (44.0 vs. 25.0%; *P* = 0.149). Similarly, no statistical difference was noticed in the incidence of grades 0–2 or 3–4 radiation pneumonitis between these two groups (*P* = 0.314; Table 2).

**TABLE 2:** Comparison of radiation-induced adverse events.

Groups	*N*	Radiation esophagitis		Radiation pneumonitis	
		**Grades 1–2**	**Grades 3–4**	**Grades 0–2**	**Grades 3–4**
HFR group	25	56.0	44.0	84.0	16
CFR group	28	75.0	25.0	92.9	7.1
*Z* value		−1.444		−1.006	
*P* value		0.149		0.314	

**HFR*, hypofractionated radiotherapy; *CFR*, conventional fractionated radiotherapy.*

### HFR Showed Similar Short-Term Efficiency Compared With CFR

Two months after radiotherapy, the complete response (CR) and partial response (PR) rate of HFR was 36 and 44%, while that of CFR was 21.4 and 53.6%, respectively. There was no statistical difference in the treatment efficiency between these two groups (*P* = 0.314; Table 3). The treatment efficiency in patients with lymphatic lesion with a diameter of ≤2 cm was significantly higher than those with lymphatic lesion with a diameter of >2 cm (*P* < 0.001; Table 4).

**TABLE 3:** Comparison of short-term efficiency between two groups.

Group	*N*	CR	PR	SD	*Z* value	*P* value
HFR group	25	36.0%	44.0%	20.0%	−1.006	0.314
CFR group	28	21.4	53.6	25.0		

**HFR*, hypofractionated radiotherapy; *CFR*, conventional fractionated radiotherapy.*

**TABLE 4:** Comparison of short-term efficiency in patients with a lymphatic metastatic lesion of different diameters.

Tumor diameter (cm)	*N*	CR	PR	SD	*Z* value	*P* value
≤2	36	36.1	55.6	8.3	−3.741	<0.001
>2	17	0.0	52.9	47.1		

### HFR Treatment Prolonged OS and Reduced Mortality

**FIGURE 1:**
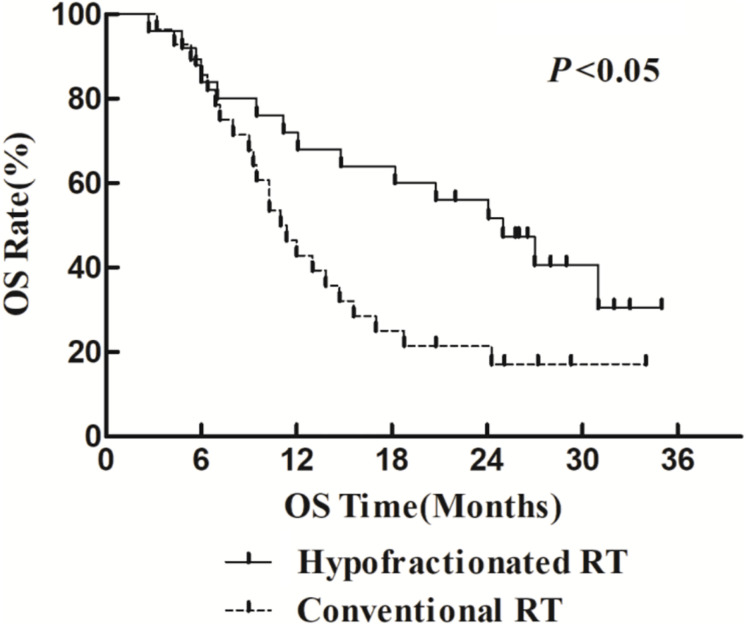
Comparison of OS between HFR group and CFR group. Patients who received HFR treatment have better OS than those treated with CFR.

**FIGURE 2:**
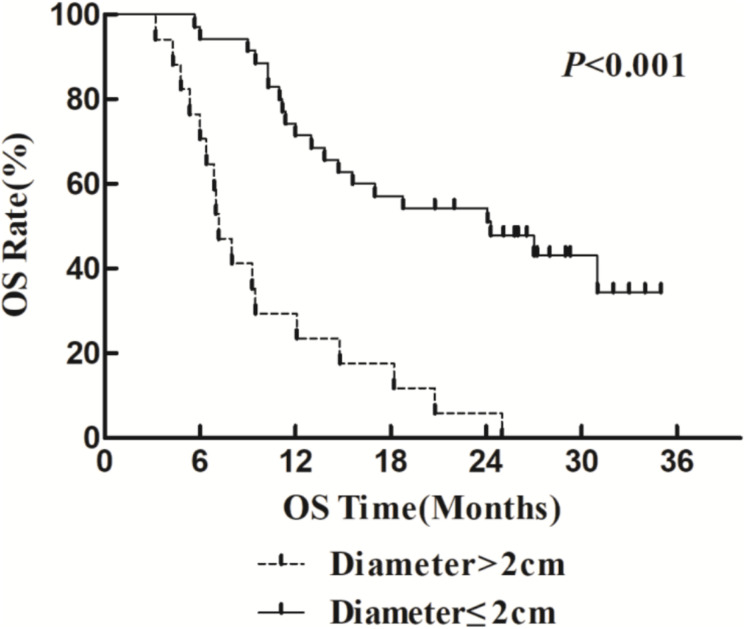
Comparison of OS in patients with various lymph node metastases. Patients with lymph node diameter ≤2 cm have better OS than those with >2 cm.

**FIGURE 3:**
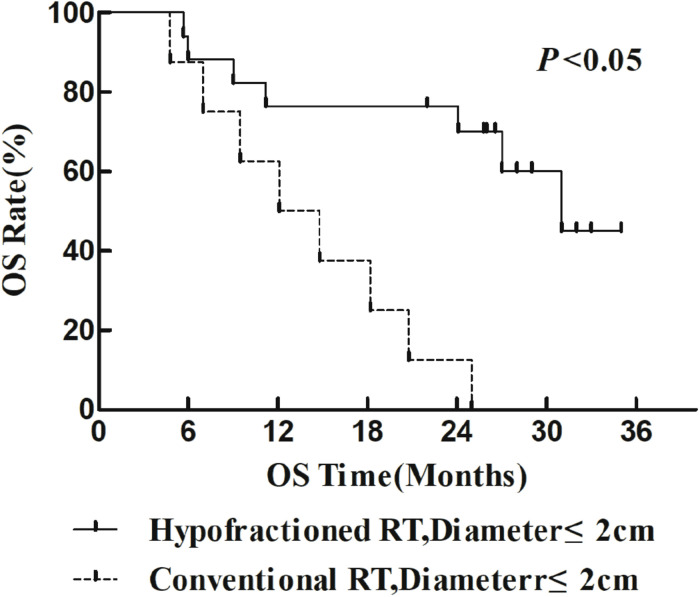
Comparison of OS between HFR group and CFR group in patients with lymph node diameter ≤2 cm.

**FIGURE 4:**
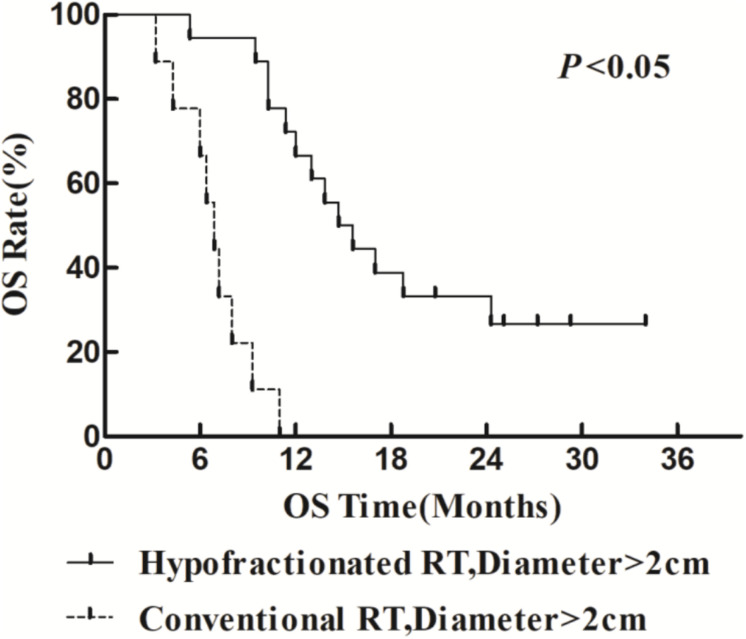
Comparison of OS between HFR group and CFR group in patients with lymph node diameter >2 cm.

The 1- and 2-year survival rate of patients was 56.6 and 35.6%, respectively (median OS, 14.7 months; 95% CI, 9.6–19.8 months). The median OS was significantly higher in the HFR group compared with that in the CFR group (24.2 months (95% CI, 16.2–32.1 months) vs. 11.8 months (95% CI, 9.2–14.4 months); *P* = 0.024; Figure 1). For the patients with lymphatic metastatic lesion ≤2 cm, the median OS was 24.1 months (95% CI, 12.0–36.1 months), which was significantly higher than those with a lymphatic metastatic lesion >2 cm, who had a median OS of 7.3 months (95% CI, 6.2–8.2 months; *P* = 0.001; Figure 2). Patients with a lymphatic metastatic lesion ≤2 cm in the HFR group showed a longer OS compared with those of the CFR group (31 vs. 7.3 months; *P* = 0.039; Figure 3). Meanwhile, patients with a lymphatic metastatic lesion >2 cm in the HFR group showed a longer OS compared with those of the CFR group (12.1 vs. 6.9 months; *P* = 0.027; Figure 4). Univariate analysis indicated that TGLN diameter (*P* < 0.001) and fractioned types (*P* = 0.028) were risk factors for the prognosis. After adjusting with the age, gender, TGLN, and fractioned types, multivariate analysis indicated that TGLN with a diameter ≤2 cm (HR = 0.108; 95% CI, 0.047–0.249) and HFR (HR = 0.236; 95% CI, 0.105–0.528) were independent risk factors for better prognosis (Table 5).

**TABLE 5:** Univariate and multivariate analyses of OS covariants.

Variable	Univariate analysis			Multivariate analysis		
	HR	95% CI	*P* value	HR	95% CI	*P* value
**Gender**						
M/F	1.326	0.639–2.749	0.449	1.371	0.626–3.002	0.429
**Age (years)**						
<60 vs. ≥60	0.832	0.393–1.761	0.832	0.537	0.247–1.164	0.115
**T staging, postoperative**						
T_3__–__4_ vs. T_1__–__2_	1.262	0.610–2.613	0.530			
**N staging, postoperative**						
N_+_ vs. N_0_	1.457	0.685–3.096	0.328			
**TNM staging, postoperative**						
I–II vs. III	1.117	0.541–2.304	0.765			
**Diameter of lymph nodes**						
≤2 vs. ≥2 cm	0.217	0.110–0.426	<0.001	0.108	0.047–0.249	<0.001
Fractioned types						
HFR vs. CFR	0.474	0.244–0.921	0.028	0.236	0.105–0.528	<0.001

**HFR*, hypofractionated radiotherapy; *CFR*, conventional fractionated radiotherapy.*

There were 38 deaths in total (71.70%) during the study period. Among the death cases, 3 cases died from TGLN recurrence in the HFR group, while in the CFR group, 12 cases died from TGLN recurrence. In each group, five cases died from metastasis to other organs. In the HFR group, one died from pulmonary embolism and one died from cardiac failure. In the CFR group, one died from pulmonary embolism. Five cases died from unknown causes in each group. The TGLN recurrence in the HFR group was significantly lower than that of the CFR group (12.0 vs. 42.9%; *P* = 0.016).

## Discussion

Surgery is commonly used for the treatment of EC, while lymphadenectomy is used for the resection of TGLN (12–15). However, TGLN metastasis after surgery is the major cause of treatment failure of EC, with an incidence of 12–80% (2–5), especially the right lymph node metastasis (16, 17). The diagnosis of TGLN metastasis is mainly relied on CT and PET-CT scans (6, 18–21), where there is a presence of a short diameter ≥0.5 cm and/or high uptake of 18F-PDG (22). In clinical practice, lymph node metastasis may induce injury of laryngeal nerve, which results in hoarseness and bucking and a poor prognosis.

There is no consensus on the treatment of TGLN metastasis after surgery. Local radiotherapy and adjuvant chemotherapy are usually used for treating TGLN metastasis. After surgery, there is a decrease in blood supply to the TGLN leading to lower sensitivity of radiotherapy. Thus, the treatment efficiency of CFR is not satisfactory. Currently, HFR has rarely been used for the treatment of TGLN metastasis, because severe tracheal and/or esophageal perforation might occur. In a previous study, Song et al. showed that a daily dose of radiation ≤5 Gy was feasible for treating patients with advanced EC with satisfactory tolerance (10). Besides, Ma et al. showed that a dose of 54–60 Gy/18–20 fractions induced no obvious adverse events and may contribute to improving efficiency. Radiotherapy was used for the management of local tumor and peripheral infiltration, and it could not control distal metastasis (23). Several studies had tried the combination of radiotherapy and chemotherapy using low-dose paclitaxel to improve the sensitivity of radiation of EC cells, which could inhibit the metastasis and improve the OS (24, 25). In this study, the major effects of paclitaxel were to sensitize radiation rather than its chemotherapy-related features.

In this study, we compared the treatment efficacy using either HFR or CFR combined with chemotherapy in patients with TGLN metastasis after surgery. The dose used in the HFR group was about 80 Gy. The incidence of radiation esophageal and pulmonary injury in the HFR group was slightly higher than those of the CFR group, but there was no statistical significance. The median OS in the patients with a lymph node diameter ≤2 cm was significantly higher than that of the counterparts with a lymph node diameter >2 cm (24.1 months (95% CI, 12.0–36.1 months) vs. 7.3 months (95% CI, 6.2–8.2 months), *P* < 0.05). This demonstrated that it would be beneficial for EC patients to receive regular thoracic CT or PET/CT in order to monitor TGLN metastasis at an early stage. The median OS in the HFR group was higher than those of the CFR group (24.2 months (95% CI, 16.2–32.1 months) vs. 11.8 months (95% CI, 9.2–14.4 months), *P* < 0.05). Patients with a lymph node metastasis of ≤2 cm in the HFR group showed a longer OS compared with those of the CFR group (31 months vs. 7.3 months, *P* = 0.039). In addition, patients with a lymph node metastasis of >2 cm in the HFR group showed a longer OS compared with those of the CFR group (12.1 vs. 6.9 months, *P* = 0.027). These demonstrated that patients with TGLN metastasis may benefit from the HFR. This was not consistent with the previous description in which a higher radiotherapy dose was required for the patients with a large tumor size (26). As previously described, pathological staging was a prognostic factor for EC (27, 28). Nevertheless, in this study, TGLN with a diameter ≤2 cm (HR = 0.108; 95% CI, 0.047–0.249) and HFR (HR = 0.236; 95% CI, 0.105–0.528) were independent risk factors for better prognosis.

There are some limitations in this study. The sample size is small because the incidence of TGLN is usually low. Studies involving a larger sample size are required to further validate the efficiency and safety of the combination of HFR and chemotherapy for the treatment of TGLN metastasis after surgery.

In conclusion, EC patients with TGLN metastasis after surgery may benefit from the combinational treatment using HFR and paclitaxel chemotherapy. This study would help clinicians to make individual treatment decisions on late-stage cancer patients.

## Data Availability Statement

The original contributions presented in the study are included in the article/supplementary material, further inquiries can be directed to the corresponding authors.

## Ethics Statement

The studies involving human participants were reviewed and approved by the Ethical Committee of Yancheng Third People’s Hospital. The patients/participants provided their written informed consent to participate in this study.

## Author Contributions

JW was responsible for data analysis and manuscript writing. JY, YJ, and DP were responsible for data collection. HZ and JLW designed the study and revised the manuscript. All authors contributed to the article and approved the submitted version.

## Conflict of Interest

The authors declare that the research was conducted in the absence of any commercial or financial relationships that could be construed as a potential conflict of interest.

## References

[1] Chen W Zheng R Baade PD Zhang S Zeng H Bray F Cancer statistics in China, 2015. *CA Cancer J Clin.* (2016) 66:115–32. 10.3322/caac.21338 26808342

[2] Altorki NK Skinner DB. Occult cervical nodal metastasis in esophageal cancer: preliminary results of three-field lymphadenectomy. *J Thorac Cardiovasc Surg.* (1997) 113:540–4. 9081100 10.1016/S0022-5223(97)70368-4

[3] Igaki H Kato H Tachimori Y Nakanishi Y. Cervical lymph node metastasis in patients with submucosal carcinoma of the thoracic esophagus. *J Surg Oncol.* (2000) 75:37–41. 10.1002/1096-9098(200009)75:1<37::aid-jso7>3.0.co;2-5 11025460

[4] Kato H Igaki H Tachimori Y Watanabe H Tsubosa Y Nakanishi Y. Assessment of cervical lymph node metastasis in the staging of thoracic esophageal carcinoma. *J. Surg. Oncol.* (2000) 74:282–5. 10962461 10.1002/1096-9098(200008)74:4<282::aid-jso8>3.0.co;2-r

[5] Luo Y Wang X Liu Y Wang C Huang Y Yu J Identification of risk factors and the pattern of lower cervical lymph node metastasis in esophageal cancer: implications for radiotherapy target delineation. *Oncotarget.* (2017) 8:43389–96. 28118614 10.18632/oncotarget.14761 PMC5522154

[6] Li X Zhao J Liu M Zhai F Zhu Z Yu F Determination of radiotherapeutic target zones for thoracic esophageal squamous cell cancer with lower cervical lymph node metastasis according to CT-images. *Oncotarget.* (2016) 7:35865–73. 27147581 10.18632/oncotarget.9094 PMC5094969

[7] Lin XD Shi XY Zhou TC Zhang WJ. [Intensity-modulated or 3-D conformal radiotherapy combined with chemotherapy with docetaxel and cisplatin for locally advanced esophageal carcinoma]. *Nan Fang Yi Ke Da Xue Xue Bao.* (2011) 31:1264–7. 21764711

[8] Cooper JS Guo MD Herskovic A Macdonald JS Martenson JA Jr. Al-Sarraf M Chemoradiotherapy of locally advanced esophageal cancer: long-term follow-up of a prospective randomized trial (RTOG 85-01). Radiation therapy oncology group. *JAMA.* (1999) 281:1623–7. 10235156 10.1001/jama.281.17.1623

[9] Ma JB Wei L Chen EC Qin G Song YP Chen XM Moderately hypofractionated conformal radiation treatment of thoracic esophageal carcinoma. *Asian Pac J Cancer Prev.* (2012) 13:4163–7. 23098423 10.7314/apjcp.2012.13.8.4163

[10] Song YP Ma JB Hu LK Zhou W Chen EC Zhang W. Phase I/II study of hypofractionated radiation with three-dimensional conformal radiotherapy for clinical T3-4N0-1M0 stage esophageal carcinoma. *Technol Cancer Res Treat.* (2011) 10:25–30. 10.7785/tcrt.2012.500176 21214285

[11] Al-Halabi H Paetzold P Sharp GC Olsen C Willers H. A contralateral esophagus-sparing technique to limit severe esophagitis associated with concurrent high-dose radiation and chemotherapy in patients with thoracic malignancies. *Int J Radiat Oncol Biol Phys.* (2015) 92:803–10. 26104934 10.1016/j.ijrobp.2015.03.018

[12] Fang Q Han YT Wang SX Ren GG Peng L Xiao WG [Clinical outcomes and selection conditions of three-field lymph node dissection for thoracic esophageal squamous cell carcinoma]. *Zhonghua Zhong Liu Za Zhi.* (2012) 34:212–5. 22780977 10.3760/cma.j.issn.0253-3766.2012.03.012

[13] Li B Xiang J Zhang Y Li H Zhang J Sun Y Comparison of Ivor-Lewis vs sweet esophagectomy for esophageal squamous cell carcinoma: a randomized clinical trial. *JAMA Surg.* (2015) 150:292–8. 25650816 10.1001/jamasurg.2014.2877

[14] Mao Y Yang D Gao S Xue QH He J. Consensus and controversies of surgical approach selection in the treatment for thoracic esophageal cancers. *Zhonghua Wei Chang Wai Ke Za Zhi.* (2016) 19:961–4. 27680059

[15] Yang X Zhan C Sun F Chen L Shi M Jiang W [Efficacy comparison of sweet versus Ivor-Lewis esophagectomy in the treatment of middle-lower esophageal squamous cell carcinoma]. *Zhonghua Wei Chang Wai Ke Za Zhi.* (2016) 19:979–84. 27680063

[16] Mao YS He J Dong JS Cheng GY Sun KL Liu XY Comparison of the results of lymph node dissection via left versus right thoracotomy. *Zhonghua Zhong Liu Za Zhi.* (2012) 34:296–300. 22781044 10.3760/cma.j.issn.0253-3766.2012.04.013

[17] Xue HC Wu CR Zhang ZB Zhu ZH Ma ZK Lin AM. Right para-tracheal triangle lymphadenectomy for esophageal carcinoma. *Zhonghua Zhong Liu Za Zhi.* (2003) 25:397–400. 12921576

[18] Fencl P Belohlavek O Harustiak T Zemanova M. FDG-PET/CT lymph node staging after neoadjuvant chemotherapy in patients with adenocarcinoma of the esophageal-gastric junction. *Abdom Radiol.* (2016) 41:2089–94. 10.1007/s00261-016-0820-x PMC5059406 27405645

[19] Foley KG Lewis WG Fielding P Karran A Chan D Blake P N-staging of oesophageal and junctional carcinoma: is there still a role for EUS in patients staged N0 at PET/CT? *Clin Radiol.* (2014) 69:959–64. 24916652 10.1016/j.crad.2014.04.023

[20] Goel R Subramaniam RM Wachsmann JW. PET/Computed tomography scanning and precision medicine: esophageal cancer. *PET Clin.* (2017) 12:373–91. 28867110 10.1016/j.cpet.2017.05.001

[21] Yap WK Chang YC Hsieh CH Chao YK Chen CC Shih MC Favorable versus unfavorable prognostic groups by post-chemoradiation FDG-PET imaging in node-positive esophageal squamous cell carcinoma patients treated with definitive chemoradiotherapy. *Eur J Nucl Med Mol Imaging.* (2018) 45:689–98. 29188300 10.1007/s00259-017-3901-3

[22] Zhao KL Ma JB Liu G Wu KL Shi XH Jiang GL. Three-dimensional conformal radiation therapy for esophageal squamous cell carcinoma: is elective nodal irradiation necessary? *Int J Radiat Oncol Biol Phys.* (2010) 76:446–51. 20004527 10.1016/j.ijrobp.2009.02.078

[23] Denham JW Burmeister BH Lamb DS Spry NA Joseph DJ Hamilton CS Factors influencing outcome following radio-chemotherapy for oesophageal cancer. The trans tasman radiation oncology group (TROG). *Radiother Oncol.* (1996) 40:31–43. 10.1016/0167-8140(96)01762-8 8844885

[24] Jeremic B Milicic B Acimovic L Milisavljeviæ S. Concurrent hyperfractionated radiotherapy and low-dose daily carboplatin/paclitaxel in patients with early-stage (I/II) non-small-cell lung cancer: long-term results of a phase II study. *J Clin Oncol.* (2005) 23:6873–80. 16192579 10.1200/JCO.2005.22.319

[25] Tu L Sun L Xu Y Wang Y Zhou L Liu Y Paclitaxel and cisplatin combined with intensity-modulated radiotherapy for upper esophageal carcinoma. *Radiat Oncol.* (2013) 8:75. 10.1186/1748-717x-8-75 23531325 PMC3622578

[26] MacCarthy P Isaac P Frost G Stokes G. Clinical dose-response studies with guanfacine (BS 100-141), a new antihypertensive agent. *Clin Exp Pharmacol Physiol.* (1978) 5:187–90. 348364 10.1111/j.1440-1681.1978.tb00669.x

[27] Blackham AU Sm HN Schell MJ Jin W Gangi A Almhanna K Recurrence patterns and associated factors of locoregional failure following neoadjuvant chemoradiation and surgery for esophageal cancer. *J Surg Oncol.* (2018) 117:150–9. 10.1002/jso.24808 28833197 PMC7771345

[28] Valmasoni M Pierobon ES De Pasqual CA Zanchettin G Moletta L Salvador R Esophageal cancer surgery for patients with concomitant liver cirrhosis: a single-center matched-cohort study. *Ann Surg Oncol.* (2017) 24:763–9. 27704371 10.1245/s10434-016-5610-8

